# *Desulfonatronobacter acetoxydans* sp. nov.,: a first acetate-oxidizing, extremely salt-tolerant alkaliphilic SRB from a hypersaline soda lake

**DOI:** 10.1007/s00792-015-0765-y

**Published:** 2015-06-18

**Authors:** D. Y. Sorokin, N. A. Chernyh, M. N. Poroshina

**Affiliations:** Winogradsky Institute of Microbiology, Russian Academy of Sciences, Prospect 60-let Octyabrya 7/2, 117811 Moscow, Russia; Department of Biotechnology, Delft University of Technology, Delft, The Netherlands; Skryabin Institute of Biochemistry and Physiology of Microorganisms, Russian Academy of Sciences, Puschino, Russia

**Keywords:** Soda lakes, Haloalkaliphilic, Acetate oxidation, Sulfate-reducing bacteria (SRB), *Desulfobacteracea*

## Abstract

Recent intensive microbiological investigation of sulfidogenesis in soda lakes did not result in isolation of any pure cultures of sulfate-reducing bacteria (SRB) able to directly oxidize acetate. The sulfate-dependent acetate oxidation at haloalkaline conditions has, so far, been only shown in two syntrophic associations of novel *Syntrophobacteraceae* members and haloalkaliphilic hydrogenotrophic SRB. In the course of investigation of one of them, obtained from a hypersaline soda lake in South-Western Siberia, a minor component was observed showing a close relation to *Desulfonatronobacter acidivorans*—a “complete oxidizing” SRB from soda lakes. This organism became dominant in a secondary enrichment with propionate as *e*-donor and sulfate as *e*-acceptor. A pure culture, strain APT3, was identified as a novel member of the family *Desulfobacteraceae*. It is an extremely salt-tolerant alkaliphile, growing with butyrate at salinity up to 4 M total Na^+^ with a pH optimum at 9.5. It can grow with sulfate as *e*-acceptor with C_3_–C_9_ VFA and also with some alcohols. The most interesting property of strain APT3 is its ability to grow with acetate as *e*-donor, although not with sulfate, but with sulfite or thiosulfate as *e*-acceptors. The new isolate is proposed as a new species *Desulfonatronobacter acetoxydans*.

**Electronic supplementary material:**

The online version of this article (doi:10.1007/s00792-015-0765-y) contains supplementary material, which is available to authorized users.

## Introduction

During the last two decades, intensive microbiological and, recently, also molecular ecology investigation of naturally occurring saline, alkaline (soda) lakes resulted in a wealth of information on functional structure of natronophilic microbial communities (last reviewed by Sorokin et al. 2014a). In particular, the microbial sulfur cycle received much attention as the most active in soda lakes (Sorokin et al. 2011). Relatively, high rates of sulfidogenesis have been detected in anaerobic sediments even in hypersaline soda lakes and the actual presence of SRB was confirmed by the analysis of both the key functional genes of the dissimilatory sulfite reductase (*dsr*AB) and, in some cases, also by the 16S rRNA gene analysis (Scholten et al. 2005; Foti et al. 2007, 2008; Mesbah and Wiegel 2012). Most of the SRB isolates and clones in soda lakes belong to lithotrophic members of the order *Desulfovibrionales* (reviewed by Sorokin et al. 2011). Despite that molecular analysis demonstrated a presence of several clusters apparently belonging to the “complete oxidizing” SRB in the family *Desulfobacteraceae*, so far, only a single haloalkaliphilic SRB, described as *Desulfonatronobacter acidivorans*, has been found in soda lakes (Sorokin et al. 2012a). Although it oxidized several volatile fatty acids (VFA) completely to CO_2_, no growth was observed with externally provided acetate. Until now, the acetate oxidation at sulfate-reducing conditions in soda lakes has only been demonstrated in two syntrophic associations of reverse acetogenic members within the clostridial family *Syntrophomonadaceae* and lithotrophic SRB. At low salt concentrations, the association included ‘*Candidatus* Contubernalis alkalaceticum’ and *Desulfonatronum cooperativum* (Zhilina et al. 2005), while ‘*Candidatus* Syntrophonatronum acetioxidans’/*Desulfonatronospira* sp. ASO3-2 were able to oxidize acetate in nearly saturated soda brines (Sorokin et al. 2014b). During the investigation of the latter at different stages of purification, a presence of a minor SRB component in the enrichment at 3 M total Na^+^ had been detected both in 16S-rRNA gene and *dsr*AB-targeted DGGE analyses (KF588528 and KF835258, respectively). Both sequences clustered with *Desulfonatronobacteracidivorans*, thus hinting at the presence of a “complete oxidizing” SRB in the acetate-oxidizing syntrophic culture. The present paper describes isolation and properties of this organism.

## Methods

### Isolation source

The source for the isolation was a syntrophic enrichment culture oxidizing acetate with sulfate as the *e*-acceptor at pH 10 and 3 M Na^+^ inoculated with anaerobic sediments from the hypersaline soda lake Bitter-1 in Kulunda Steppe, Altai, Russia (Sorokin et al. 2014b).

### Enrichment, isolation and cultivation conditions

Anaerobic sub-enrichment with propionate as *e*-donor (10 mM) and sulfate (20 mM) was performed from the syntrophic acetate-oxidizing mixed culture obtained from sulfidic sediments of hypersaline soda lake Bitter-1 in Kulunda Steppe, Altai, Russia (Sorokin et al. 2014b) and maintained at 2–3 M total Na^+^, pH 10 and 30 °C. The basal sodium carbonate-based mineral medium containing 3 M total Na^+^ was made by 1:1 mixing of the media containing 2 and 4 M total Na^+^, pH 10, as described previously (Sorokin et al. 2011b). Routine cultivation was performed in Hungate tubes with 10 ml medium and for the large-scale cultivation—in 100–500 ml bottles capped with butyl rubber stoppers and filled to 75 % volume. The sub-enrichment with propionate and sulfate was first done in 1:10 dilution which resulted in the formation of 9 mM sulfide in 2 months period. Next, the culture was transferred into a new medium containing 2 M total Na^+^ at 1:100 dilution. After 2–3 successful transfers, the enrichment was serially diluted up to 10^−10^ using propionate or butyrate as *e*-donors and sulfate or thiosulfate as *e*-acceptors. A combination of butyrate + thiosulfate resulted in faster growth and higher cell density. Therefore, it was used for the final isolation of a pure culture. Since the colony formation was not achieved, the final purification was done by several rounds of serial dilutions in liquid medium. The final purity of the isolate (strain APT3) was checked by microscopy, by the absence of growth on rich media (pyruvate + yeast extract) without electron acceptors and by sequencing. The pH dependence of growth and activity of washed cells was examined at 2 M total Na^+^ using 0.1 M HEPES/NaCl/NaHCO_3_ for the pH 6–8 and a mixture of sodium bicarbonate/sodium carbonate containing 0.1 M NaCl for the pH 8.5–11. The final pH values were taken to indicate a suitable range for growth and activity. To study the influence of Na^+^ concentration on growth and activity, sodium carbonate-based buffers with pH 9.5 containing 0.3–4.0 M of total Na^+^ were mixed in different proportions.

### Analyses

Sulfide was precipitated in 10 % (w/v) Zn acetate and analyzed by the methylene blue method after separation from the supernatant (Trüper and Schlegel 1964). Thiosulfate and sulfite were determined after the removal of ZnS by acidic iodimetric titration. Acetate and butyrate concentrations were measured by HPLC anionic chromatography, as described previously (Sorokin et al. 2012a). The cell growth was monitored by measuring OD_600_. Membrane polar lipids for the PLFA analysis were extracted from freeze-dried biomass by acidic methanol and their fatty acid composition examined with GC–MS according to Zhilina et al. (1997). For the total phospholipid identification, wet biomass extraction protocol was used and the lipids were identified by two-dimensional TLC as described previously (Sorokin et al. 2012a). Respiratory lipoquinones were analyzed in cold acetone extract first by TLC (Collins 1985) and then a major UV-absorbing band was eluted and subjected to tandem mass spectrometry (LCG Advantage Max) with chemical ionization at atmospheric pressure and the quinones were identified by ionic mass by HPLC–MS. Phase-contrast photomicrographs were obtained with a Zeiss Axioplan Imaging 2 microscope (Göttingen, Germany).

### Genetic and phylogenetic analysis

Isolation of genomic DNA and determination of the G + C content of the DNA from pure cultures were performed according to Marmur (1961) and Marmur and Doty (1962). For molecular analysis, the DNA was extracted from the cells using the UltraClean Microbial DNA Isolation kit (MoBio Laboratories Inc., Carlsbad, CA, USA) following the manufacturer’s instructions. The nearly complete 16S rRNA gene was obtained with general bacterial primers 11f-1492r (Lane 1991). The *dsr*AB genes were amplified with the primer pair DSR1F/DSR4R [AC(GC)CACTGGAAGCACG/GTGTAGCAGTTACCGCA] according to Wagner et al. (1998). The PCR mixture was incubated for 5 min at 94 °C, followed by 34 cycles of 20 s at 93 °C, 45 s 55 °C, and 190 s at 72 °C, with the final extension at 72 °C for 10 min. The PCR products were purified using the Qiagen Gel Extraction Kit (Qiagen, The Netherlands). The sequences were first compared to all sequences stored in GenBank using the BLAST algorithm and were consequently aligned using CLUSTAL W. The evolutionary history was inferred using the Neighbor-Joining method and the trees were constructed using the MEGA-6 package (Tamura et al. 2013).

## Results and discussion

### Cell morphology of strain APT3

**Fig. 1:**
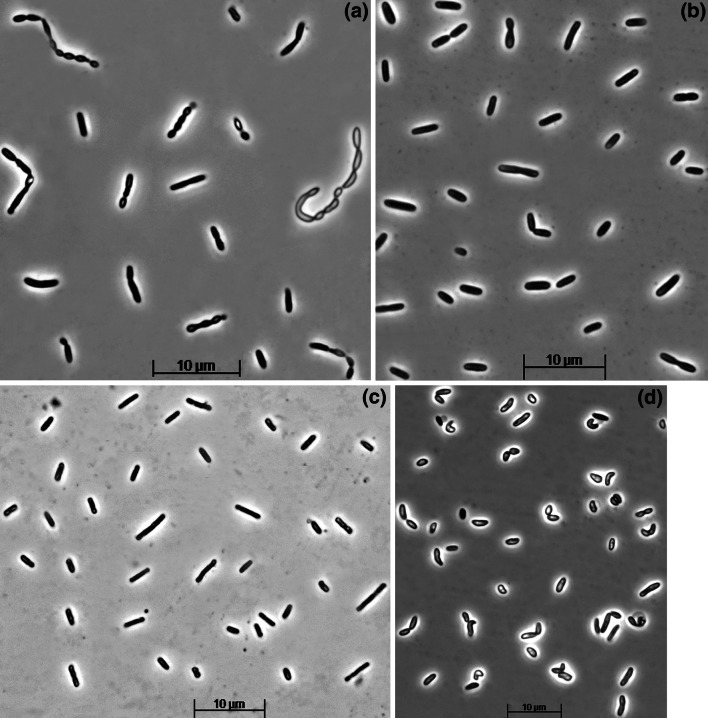
Cell morphology (phase contrast microphotographs) of strains APT3 grown at 2 M Na^+^, pH 9.5 with thiosulfate as *e*-acceptor and propionate (**a**), butyrate (**b**), acetate (**c**) or caprylate (**d**) as *e*-donor

The cell morphology of strain APT3 significantly varied with the growth conditions, but, in general, it can be defined as non-motile rod-shaped (Fig. 1).

### Identification of strain APT3 and chemotaxonomy

**Fig. 2:**
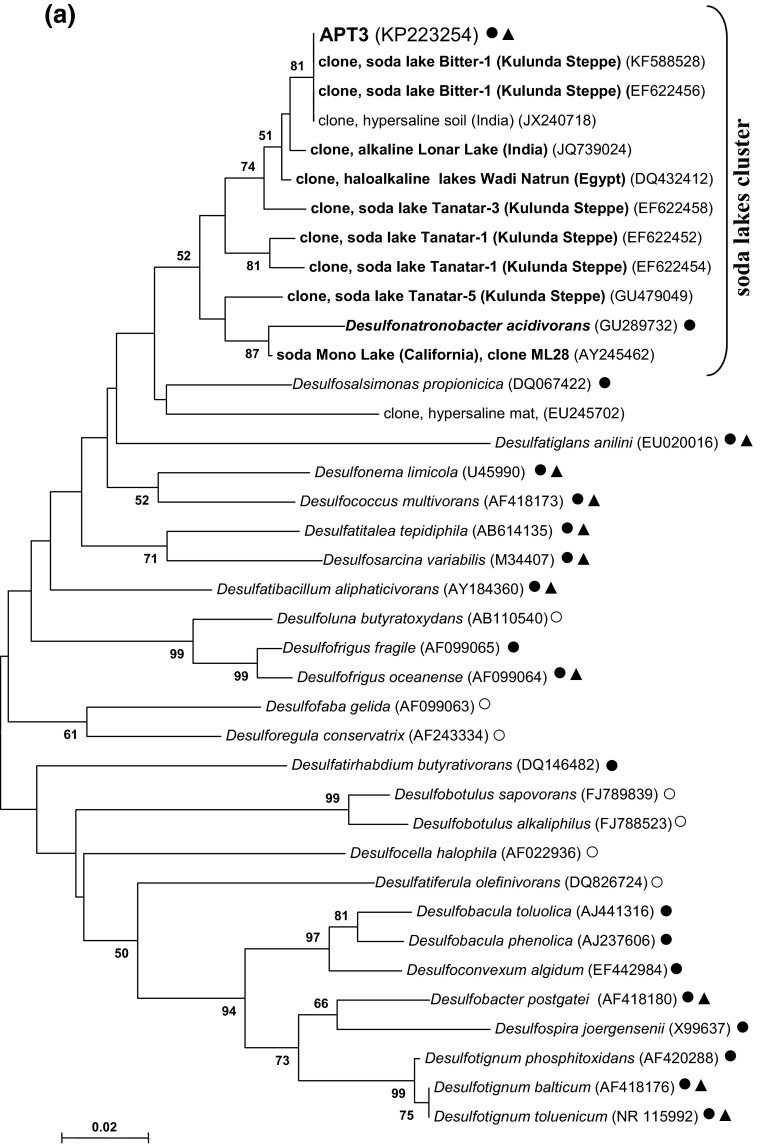

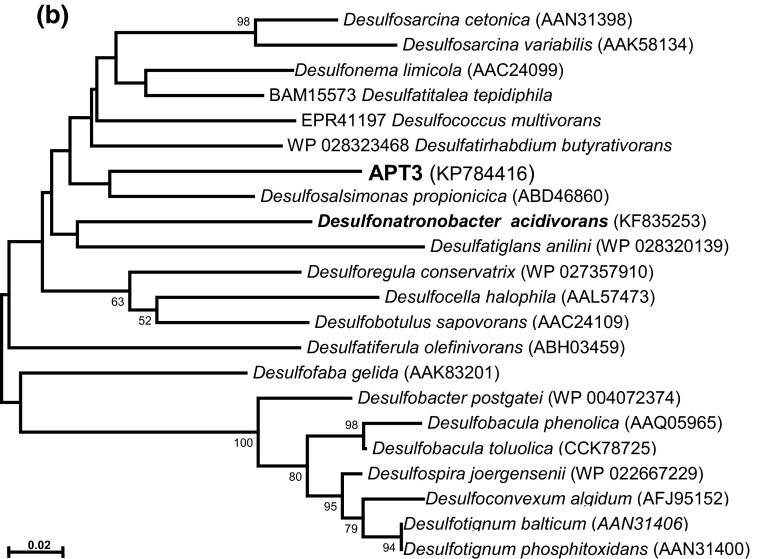

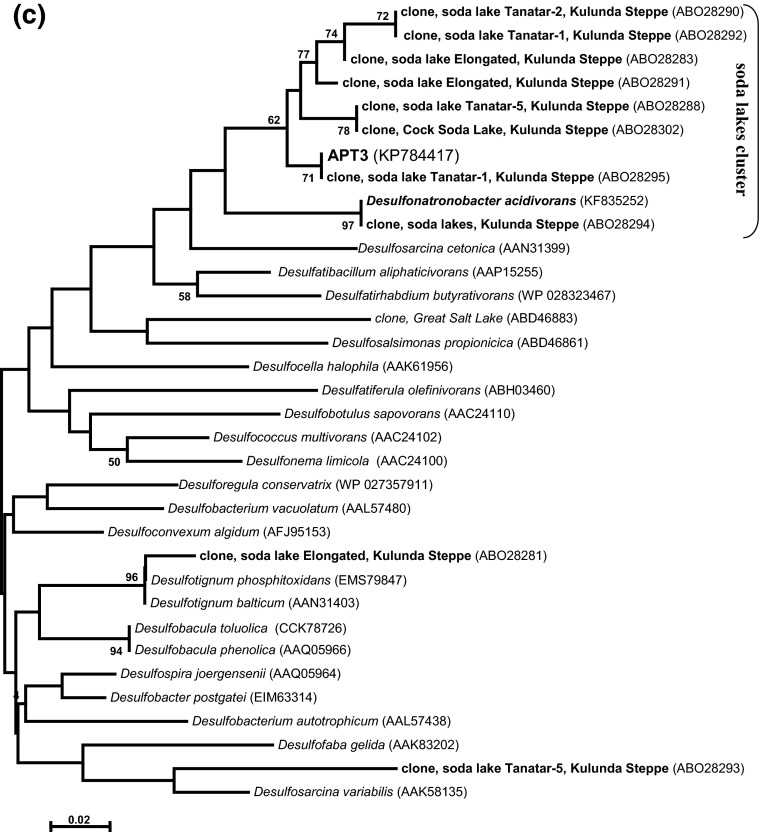
Phylogenetic position of strain APT3 based on 16S rRNA gene (**a**), dsrA (**b**) and dsrB (**c**) sequence analysis. The bootstrap values above 50 % from 1000 replicates are shown next to the branches. The evolutionary distances were computed using the neighbour joining and maximum likelihood methods and are in the units of the number of base substitutions per site. Symbols in (**a**): *open circle*, incomplete oxidizers; *closed circle*, complete oxidizers; *closed triangle*, acetate oxidizers. Clones and pure cultures obtained from alkaline saline lakes are in *bold*

According to the 16S rRNA gene analysis, the isolate belonged to the family *Desulfobacteraceae*—the deltaproteobacterial family mostly harboring “complete oxidizing” SRB genera (Kuever et al. 2005; Kuever 2014). The sequence had 100 % match to the sequence of DGGE band obtained from the source syntrophic acetate-oxidizing culture (Sorokin et al. 2014b), which confirmed that the pure culture represented the minor component detected in the source mixed culture. The closest cultured relatives are haloalkaliphilic *Desulfonatronobacter acidivoran*s (96 % sequence similarity) and halophilic *Desulfosalsimonas propionicica* (95 % sequence similarity). APT3 and *Desulfonatronobacter* clearly formed a separate “soda lake” cluster within the *Desulfobacteraceae* together with several clones from Kulunda soda lakes and other soda lakes (Fig. 2a). The fact of direct detection with general primers indicates that this particular type of SRB might be important for the sulfur cycle in soda lakes.

Phylogenetic analysis of the functional molecular marker *dsr*AB placed APT3 as a separate branch within the *Desulfobacteraceae* (Fig. 2b, c). DsrB clustering of APT3 was more consistent with the ribosomal gene-based phylogeny and also showed a presence of the “soda lakes cluster”, while the DsrA-based clustering was slightly different. However, in both cases, the boot-strap values were mostly below 50 % indicating that the branching was ambiguous in both algorithms used (NBJ and ML). Also, DsrA can only be compared with cultured SRB, since only DsrB was studied for uncultured SRB in soda lakes. The latter also shows that, most probably, there are two more groups of potential acetate-oxidizing SRB in soda lakes, associated with the genera *Desulfotignum* and *Desulfosarcina*.

The PLFA profile of strain APT3 was similar, but not identical, to that of the closest relative, *D. acidivorans*. Both organisms had two major species in the profile, 18:1ω7 and 16:0, but differed in the subdominants (Supplementary Table S1). The dominant identifiable phospholipids included phosphatidylglycerol, phosphatidyldiglycerol, phosphatidylethanolamine and phosphatidylcholine. Apart, 3 unidentified phospholipids were present (Supplementary Fig. S1). The major identified respiratory lipoquinone was MK-9, in contrast to MK-8 present in *D. acidivorans*.

### Physiological characteristics of strain APT3

**Fig. 3:**
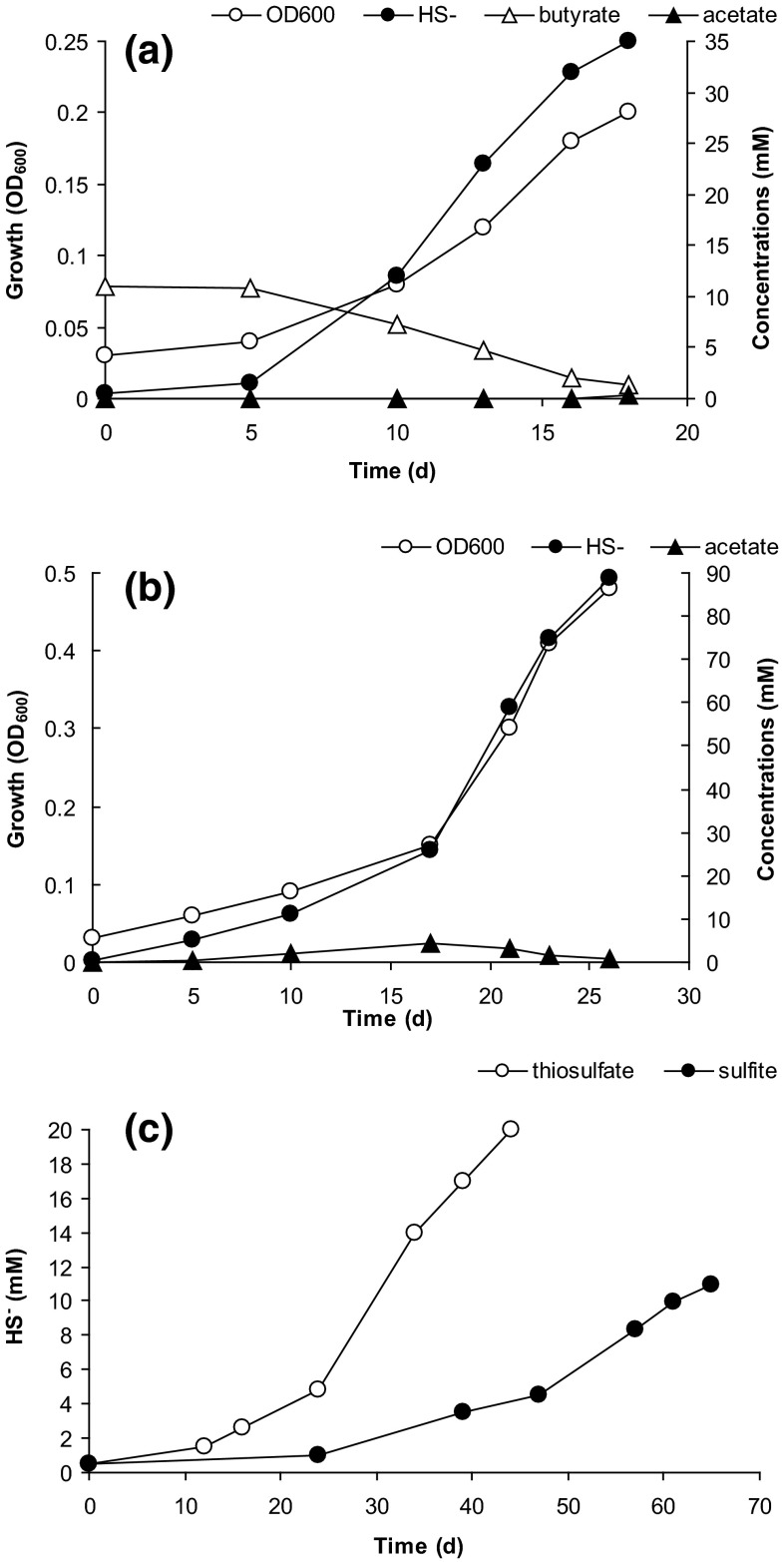
Growth dynamics of strain APT3 with (**a**) butyrate + thiosulfate, (**b**) caprylate + thiosulfate (pH 9.5 and 2 M Na^+^) and (**c**) with acetate + sulfite (*closed circles*) or thiosulfate (*open circles*) at pH 9.5 and 1 M total Na^+^. The data represent mean values from duplicate cultures with standard deviations below 15 %

**Fig. 4:**
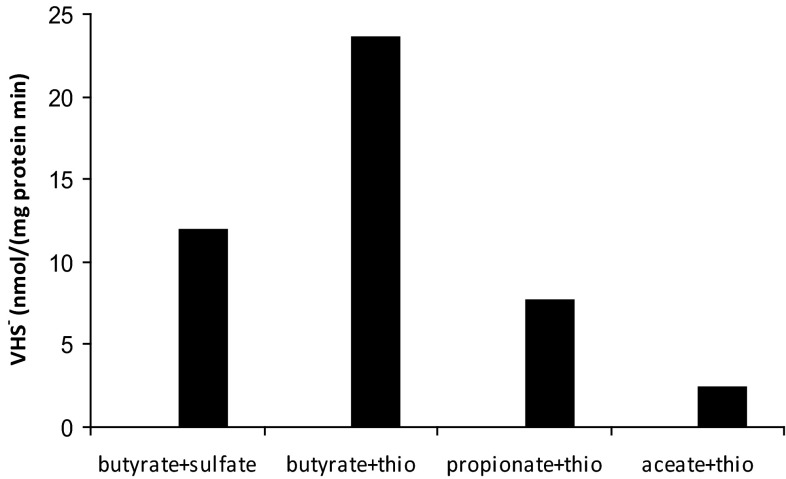
Sulfide production activity in washed cells of strain APT pre-grown with butyrate and thiosulfate

APT3 was originally isolated with propionate as *e*-donor and sulfate as *e*-acceptor. With sulfate, it was also able to use other VFA from C_4_ to C_9_ with the highest growth rate on butyrate. Apart from that, it also can grow with i-butyrate, pyruvate, lactate, and alcohols, including EtOH, 1-BuOH and 2-BuOH. Growth on acetate with sulfate as *e*-acceptor was not observed. However, when sulfate was replaced for sulfite or thiosulfate, APT3 was able to grow with acetate, albeit much slower, than with other e-donors. In anyway, this makes APT3 a first example of culturable haloalkaliphilic acetate-oxidizing SRB. The growth dynamics with three different *e*-donors is shown in Fig. 3. Acetate was not produced during oxidation of butyrate and was only a marginal intermediate during oxidation of caprylate, confirming the complete oxidation pathway of VFA to CO_2_. When washed cells of APT3 pregrown with butyrate + thiosulfate were tested on their ability to reduce sulfate and thiosulfate with butyrate and potential intermediates (propionate and acetate), the activity was in the following order: butyrate > propionate > acetate, which corresponded well to the growth rate difference with these *e*-donors (Fig. 4).

**Fig. 5:**
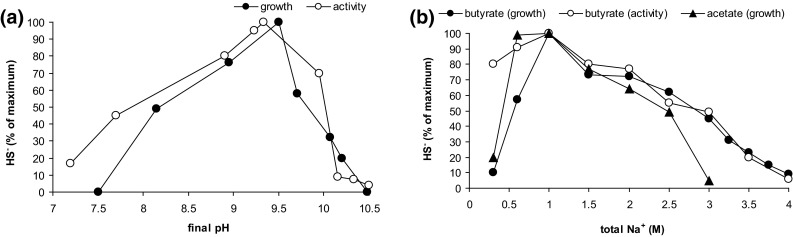
Influence of pH at 2 M Na^+^ on growth and sulfidogenic activity of washed cells of strain APT3 with butyrate (**a**) and influence of sodium carbonate concentration at pH 9.5 (**b**) on growth with butyrate or acetate and cell activity with butyrate. Thiosulfate served as the *e*-acceptor in all experiments. The *data* represent mean values from duplicate experiments with standard deviations below 12 %

Influence of pH on growth and activity of APT3 was tested with butyrate + thiosulfate at 2 M total Na^+^. The results demonstrated that, in contrast to *D. acidivorans*, APT3 is only a moderate alkaliphile with a maximum pH limit around 10 and with an optimum at 9–9.5 (Fig. 5a). On the other hand, the new isolate is remarkable in its extremely wide salt range (tested at pH 9.5), being able to grow and to produce sulfide up to soda saturation at 4 M Na^+^ (Fig. 5b). In this, APT3 represents the most salt-tolerant “complete oxidizing” SRB isolated in pure culture. With acetate as *e*-donor, the highest Na^+^ limit for growth was 2.5 M, which is in the same range as for the most salt-tolerant halophilic acetate oxidizer—*Desulfobacter halotolerans* (Brandt and Ingvorsen 1997).

In conclusion, for the first time, an alkaliphilic and extremely salt-tolerant heterotrophic acetate-oxidizing SRB, a member of the *Desulfobacteraceae*, has been obtained from a hypersaline soda lake. Its ability to oxidize acetate with sulfite and thiosulfate, but not with sulfate, as *e*-acceptor might be explained by the fact that both acetate and sulfate need ATP-dependent activation, while thiosulfate and sulfite are not. Adding to a very low energy yield of acetate-dependent sulfate reduction catabolism (ΔG^0^′ = −47 kJ/mol) (Oren 2011), growth by this reaction at double extreme conditions must be very sensitive to any extra energy saving, which is provided using sulfite/thiosulfate in contrast to sulfate.

On the basis of distinct phylogenetic, and phenotypic properties (Table 1), strain APT3 is proposed as a new species of the genus *Desulfonatronobacter* - *D. acetoxydans*.

**Table 1:** Comparative properties of strain APT3^T^ and its closest (halo)alkaliphilic relatives from the *Desulfobacteraceae* (Brandt and Ingvorsen 1997;  Kjeldsen et al. 2010; Sorokin et al. 2012a); nd, not determined

Property	APT3	*Desulfonatronobacter acidivorans*	*Desulfosalsimonas propionicica*	*Desulfobacter halotolerans*
Cell morphology	Rods	Short rods	Rods	Rods
Motility	−	+	+	+
Dominant PLFA	18:1ω7, 16:0, i14:0	18:1ω7, 16:0, 16:1ω7	nd	nd
Dominant respiratory lipoquionone	MK-9	MK-8	nd	nd
Identified membrane polar lipids	Phosphatidylglycerol, diphosphatidylglycerol phosphatidylethanolamine, phosphatidylcholine		nd	nd
Acetate as *e*-donor	+ (with thiosulfate and sulfite)	−	−	+ (with sulfate)
Other *e*-donors	C_3_–C_9_ VFA, pyruvate, lactate, i-butyrate, EtOH, BuOH, i-BuOH	C_3_–C_8_ VFA	H_2_, propionate, butyrate, i-butyrate, fumarate, lactate, EtOH, PrOH, BuOH	EtOH
Electron acceptors	Sulfate, sulfite, thiosulfate	Sulfate, thiosulfate	Sulfate, sulfite, thiosulfate	Sulfate, sulfite, thiosulfate
Salt range (optimum), M Na^+^	0.3–4.0 (1.0)	0.2–1.5 (0.6)	0.1–3.4 (1.0)	0.1–2.0 (0.2–0.3)
pH range (optimum)	8.0–10.1 (9.5)	8.5–10.6 (10.0)	6.0–8.3 (7.0)	6.2–8.1 (6.2–7.4)
G + C, mol %	53.5	54.4	54.1	49.0
Habitat	Soda lakes		Great Salt Lake	

### Description of *Desulfonatronobacter acetoxydanssp. nov*

[acet.’oxi.dan.s] L. n. *acetum*, vinegar; N. L. n. *acidumaceticum*, acetic acid; N. L. v. *oxido* (from Gr. adj. *oxys*, acid or sour and in combined words indicating oxygen), to make acid, oxidize; N.L. part. adj. *acetoxydans*, oxidizing acetate.

Cells are non-motile Gram-negative rods of variable size, depending on cultivation conditions, 0.5–1.0 × 1.0–5.0 μm, single, in pairs or in short chains. The dominant respiratory quinone is MK-9. The dominant PLFA includes 18:1ω7, 16:0 and i14:0. The dominant identified membrane phospholipids include phosphatidylglycerol, phosphatidyldiglycerol, phosphatydylethanolamine and phosphatidylcholine. Obligately anaerobic, utilizing C_3_–C_9_ VFA, i-butyrate, lactate, pyruvate, EtOH, 2-ProOH, 1-BuOH and 2-BuOH as carbon and energy with sulfate, sulfite and thiosulfate as *e*-acceptor. Acetate can serve as *e*-donor with either sulfite or thiosulfate (but not sulfate) as *e*-acceptor. The utilized *e*-donors are completely oxidized to CO_2_. Extremely salt-tolerant with a salinity range for growth from 0.3 to 4.0 M total Na^+^ (optimum at 0.6 M) and obligately alkaliphilic with a pH range for growth between 8.0 and 10.1 (optimum at pH 9–9.5). The maximum growth temperature at 2 M total Na^+^ is 43 °C (optimum 37–40 °C). The G + C content of the DNA is 53.5 mol % (*T*_m_). Isolated from sediments of the hypersaline soda lake Bitter-1 in south-western Siberia (Altai, Russia). The type strain is APT3^T^ (DSM29847 = UNIQEM U992).

## Electronic supplementary material

Supplementary material 1 (PDF 492 kb)
