# Supplementary material for: Desulfonatronobacter acetoxydans sp. nov.,: a first acetate-oxidizing, extremely salt-tolerant alkaliphilic SRB from a hypersaline soda lake
Source: Extremophiles. 2015 Jun 18;19(5):899–907. doi: 10.1007/s00792-015-0765-y (PMC4546703; doi:10.1007/s00792-015-0765-y)
Supplement: Supplementary file 1 — Supplementary material 1 (PDF 492 kb) [file 792_2015_765_MOESM1_ESM.pdf]

Supplementary Table S1. Composition of PLFA in haloalkalphilic heterotrophic SRB from soda lakes. Strain APT3 was grown at pH 9.5 and 2 M total Na^+, *Desulfonatronobacter acetioxydans* APT2 was grown at pH 10 and 0.6 M total Na^+, temperature was 30°C. The cells were harvested at late logarithmic stage. The values above 5% from the total are in bold. Only components present at the level above 0.5% are shown

FA	**APT3^T**	*Desulfonatronobacter acetioxydans* APT2^T
10:0	0.5	
12:0	1.0	
14:0	2.7	0.7
i14	**8.6**	1.1
16:0	**25.4**	**13.2**
i16:0	2.7	1.2
16:1ω7	3.3	**7.2**
18:0	1.3	1.9
i18:0	0.5	0.2
18:1ω7	**49.5**	**65.8**
11Me18:1	1.0	**6.5**
19cyc ω7	1.7	0.6

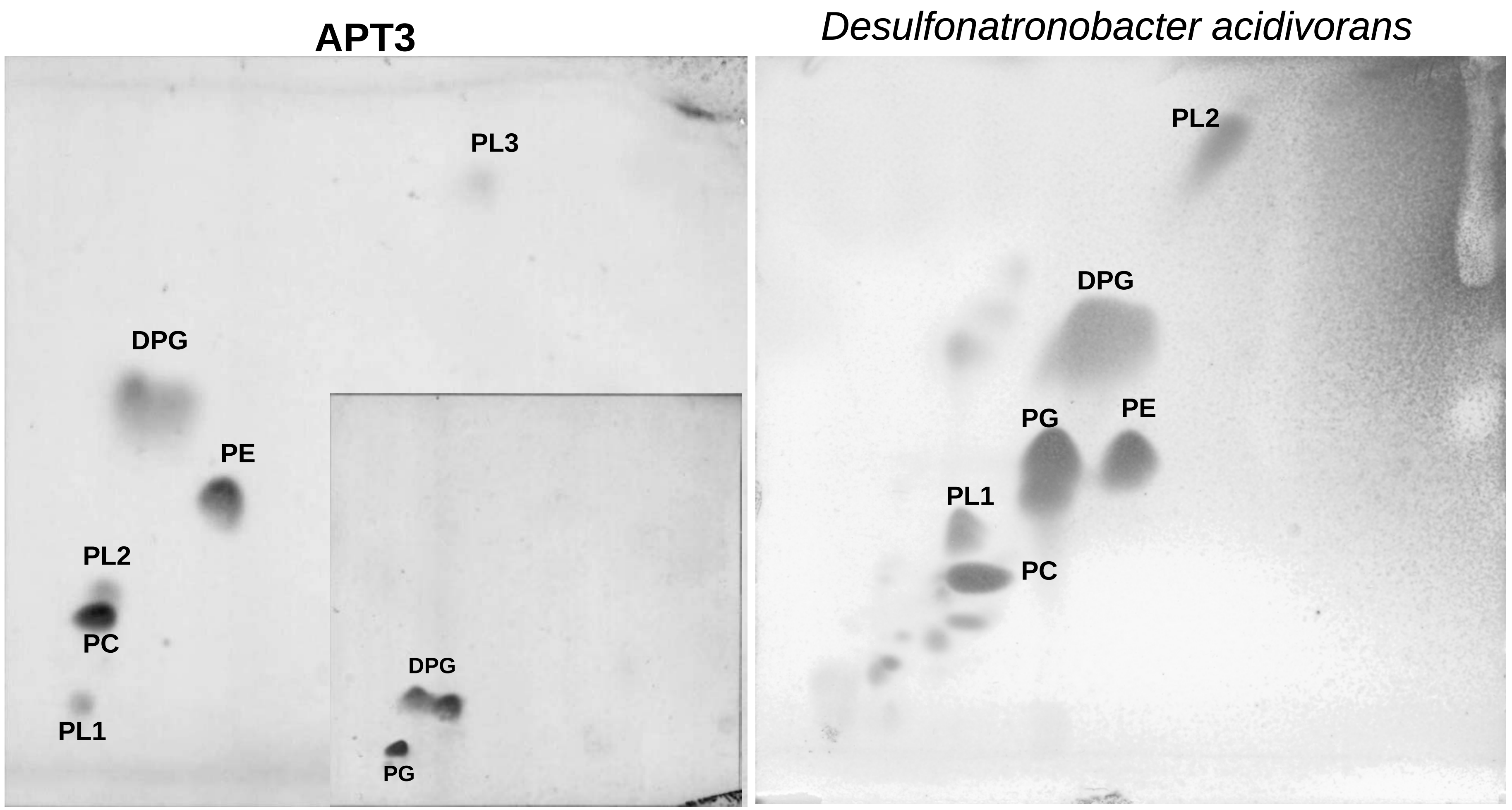

Supplementary fig S1: Membrane polar lipid profiles (2-dimensional TLC) of strain APT3 in comparison with the closest phylogenetic relative. Strains were grown with butyrate+thiosulfate at pH 10 and 0.6 M total Na^+ (*D.acidivorans*) or 2 M (APT3).
